# Zika virus vertical transmission in interferon receptor1-antagonized *Rag1*^−/−^ mice results in postnatal brain abnormalities and clinical disease

**DOI:** 10.1186/s40478-022-01351-6

**Published:** 2022-04-04

**Authors:** Clayton W. Winkler, Chad S. Clancy, Rebecca Rosenke, Karin E. Peterson

**Affiliations:** 1 Laboratory of Persistent Viral Diseases, Rocky Mountain Laboratories, National Institute of Allergy and Infectious Diseases, National Institutes of Health, 903 S. 4th Street, Hamilton, MT 59840 USA; 2 Rocky Mountain Veterinary Branch, Rocky Mountain Laboratories, National Institute of Allergy and Infectious Diseases, National Institutes of Health, Hamilton, MT 59840 USA

**Keywords:** Zika virus, Pathogenesis, Postnatal infection, Congenital brain abnormalities, Cell death, Cerebellar progenitor proliferation, Neurodevelopment

## Abstract

The mechanisms by which vertically transmitted Zika virus (ZIKV) causes postnatal brain development abnormalities and congenital disease remain poorly understood. Here, we optimized the established anti-IFNAR1 treated, *Rag1*^−/−^ (AIR) mouse model of ZIKV infection to examine the consequence of vertical transmission on neonate survival and postnatal brain development. We found that modulating the infectious dose and the frequency of anti-IFNAR1 treatment of pregnant mice (termed AIR^low^ mice) prolonged neonatal survival allowing for pathogenesis studies of brain tissues at critical postnatal time points. Postnatal AIR^low^ mice all had chronic ZIKV infection in the brain that was associated with decreased cortical thickness and cerebellar volume, increased gliosis, and higher levels of cell death in many brain areas including cortex, hippocampus and cerebellum when compared to controls. Interestingly, despite active infection and brain abnormalities, the neurodevelopmental program remained active in AIR^low^ mice as indicated by elevated mRNA expression of critical neurodevelopmental genes in the brain and enlargement of neural-progenitor rich regions of the cerebellum at a developmental time point analogous to birth in humans. Nevertheless, around the developmental time point when the brain is fully populated by neurons, AIR^low^ mice developed neurologic disease associated with persistent ZIKV infection in the brain, gliosis, and increased cell death. Together, these data show that vertically transmitted ZIKV infection in the brain of postnatal AIR^low^ mice strongly influences brain development resulting in structural abnormalities and cell death in multiple regions of the brain.

**Supplementary Information:**

The online version contains supplementary material available at 10.1186/s40478-022-01351-6.

## Introduction

Zika virus (ZIKV) is a positive-sense RNA arbovirus in the family *Flaviviridae* that recently has resurfaced across the globe and in a 2015 South American outbreak, was first associated with an increase in congenital birth defects [3, 29]. The defects are known to be associated with vertical transmission (VTx) of ZIKV from the infected mother to the fetus [46]. While not fully delineated, most infants born to ZIKV infected mothers are clinical unaffected and developed normally [36]. Of fetuses exposed to ZIKV in utero, it is estimated ~ 20 to 30% become infected which can result in fetal loss (4–7%) or manifestation of a spectrum disorder of fetal and neonatal defects (5–14%) collectively termed congenital Zika syndrome (CZS). The remainder of neonates from infected pregnancies are asymptomatic after birth but may go on to develop variable sequelae. As ZIKV is a neurotropic virus [48], the most severe of these defects afflict the central nervous system (CNS) and can result in cerebral cortex, cerebellar and corpus callosum abnormalities and in extreme cases microcephaly [30, 63]. These abnormalities can sometimes be detected in utero [35] and are thought to be the result of multiple factors including virus-associated depletion and dysregulation of neural progenitor cells [12, 27, 43, 58, 71] and CNS inflammation [41]. However, a portion of infants born to infected mothers show no overt signs of CZS until birth or thereafter [37, 60] suggesting a postnatal phase or altogether different disease can manifest from in utero ZIKV infection. The pathogenesis of this late-onset congenital syndrome has not been well studied. Furthermore, there is evidence that postnatal ZIKV CNS infection can persist for months in children following VTx [1, 7, 40]. This persistent infection may drive postnatal disease and influence later neurodevelopment which carries on in humans for many years postnatally [20].

The mechanisms by which ZIKV infection causes CNS abnormalities are still not fully understood. Animal models have been useful to understand some fundamental processes of ZIKV-induced CNS abnormalities [10, 19, 27, 38, 43, 52, 71]. However, these studies can be difficult as ZIKV does not readily infect wildtype mice, limiting the development of VTx models. Indeed, many models use high doses of virus or direct infection of fetal tissues including the placenta, amniotic fluid or the fetus itself. While these studies are informative, the dose and method of inoculation can overwhelm or circumvent the physical and immunological pressures that influence virus transmission across an intact placenta [18, 26]. These pressures can influence the spread of the virus within the fetus and subsequent disease [31]. Additionally, many studies using vertically transmitted ZIKV focus on ZIKV pathogenesis at birth or during fetal neurodevelopment [10, 27, 71]. Although this period is critical for establishing brain structure [62], postnatal neurodevelopment accounts for the majority of brain volume and density in both rodents [50] and humans [5] and critical neuronal connections related to learning and motor function are established during this time [65]. Few studies examine postnatal timepoints and largely report minimal, nonprogressive disease in the absence of active postnatal CNS infection [21, 43]. One study did identify severe CNS hypoplasia at post-natal timepoints [38], but this was done following neonatal infection, in effect modeling post-natal ZIKV infection, not VTx. Thus, a model of vertically transmitted, persistent infection is required to better understand the effect of ZIKV VTx on postnatal neurodevelopment.

Immunocompetent mice are resistant to ZIKV infection and VTx [24, 43, 57]. We and others have demonstrated that interferon (IFN) [16, 24] and adaptive immune responses [13, 68] underpin this resistance. Thus, we treated *Rag1*^−/−^ mice, which are deficient in T- and B-cells [34], with anti-IFNAR1 blocking antibody that inhibits signaling through the IFN-α/β receptor (subsequently called AIR mice) to reduce their resistance to ZIKV infection. AIR mice have been shown to consistently transmit virus to their young and have been useful for studying mechanisms of VTx, and the placental immune response [67, 70]. We have previously demonstrated that ZIKV VTx in the AIR model occurs nears birth and that infected pups have no obvious CNS abnormalities at that time point suggesting these mice could be useful to study postnatal disease. *Rag1* transcripts are expressed in the mouse brain at low levels during development [8, 55]. However, further examination of brain structure determined that *Rag1* knockout did not significantly interfere with neurodevelopment [34]. Although T-cells have been linked to behavior and memory in mice [22, 47, 73], these are characteristic of adult cognition and not directly related to postnatal neurodevelopment. Thus, *Rag1*^*−/−*^ mice could be ideal to study postnatal ZIKV infection and pathogenesis, as *Rag1* does not influence fetal or postnatal brain development.

Initial studies using infected AIR mice demonstrate that neonates can survive to at least postnatal day 2 (P2) [70]. However, the consequence of fetal infection on postnatal survival has not been studied in this model. The goal of this work was to establish whether postnatal AIR mice had active CNS infection, displayed any CNS structural abnormalities or developed disease. Using the standard AIR protocol, neonates would rarely survive to P5. Ideally, mice would survive to at least P7 as this timepoint is neurodevelopmentally analogous to the early postnatal period in humans [50]. We found that decreasing the anti-IFNAR1 treatment and the infectious dose of ZIKV in pregnant dams resulted in increased survival of neonates and allowed analysis of postnatal development. Pups from dams treated with this AIR low-dose protocol (AIR^low^) had persistent ZIKV CNS infection associated with structural abnormalities. Further analysis showed differences in glial activation, developmental gene expression and cerebellar neural progenitor distribution between pups born from AIR^low^ dams versus pups from naïve or IgG-treated controls.

## Materials and methods

### Aim, design and setting of the study

The goal of this study was to examine the effect of vertically transmitted ZIKV on CNS structure and development. Thus, we infected pregnant mice following various treatments (described below) and followed the resulting pups for survival and development of CNS pathology. All mouse experiments were performed under the approval of the Rocky Mountain Laboratories Institutional Animal Care and Use Committee and adhered to the National Institutes of Health guidelines and ethical policies under protocols 2018-001E and 2020-082E.

### Virus

The previously described Zika virus Paraiba strain [10] isolated from a human clinical case in 2015 was used for all experiments. Virus was a kind gift of Dr. Stephan Whitehead (National Institutes of Health). Working virus stocks were generated and plaque forming unit (PFU) titers of stocks were determined as previously described [67]. All stocks were frozen at − 80 °C for future use. Virus stocks underwent no more than three passages from the founder stock.

### Generation and infection of experimental mice

**Fig. 1:**
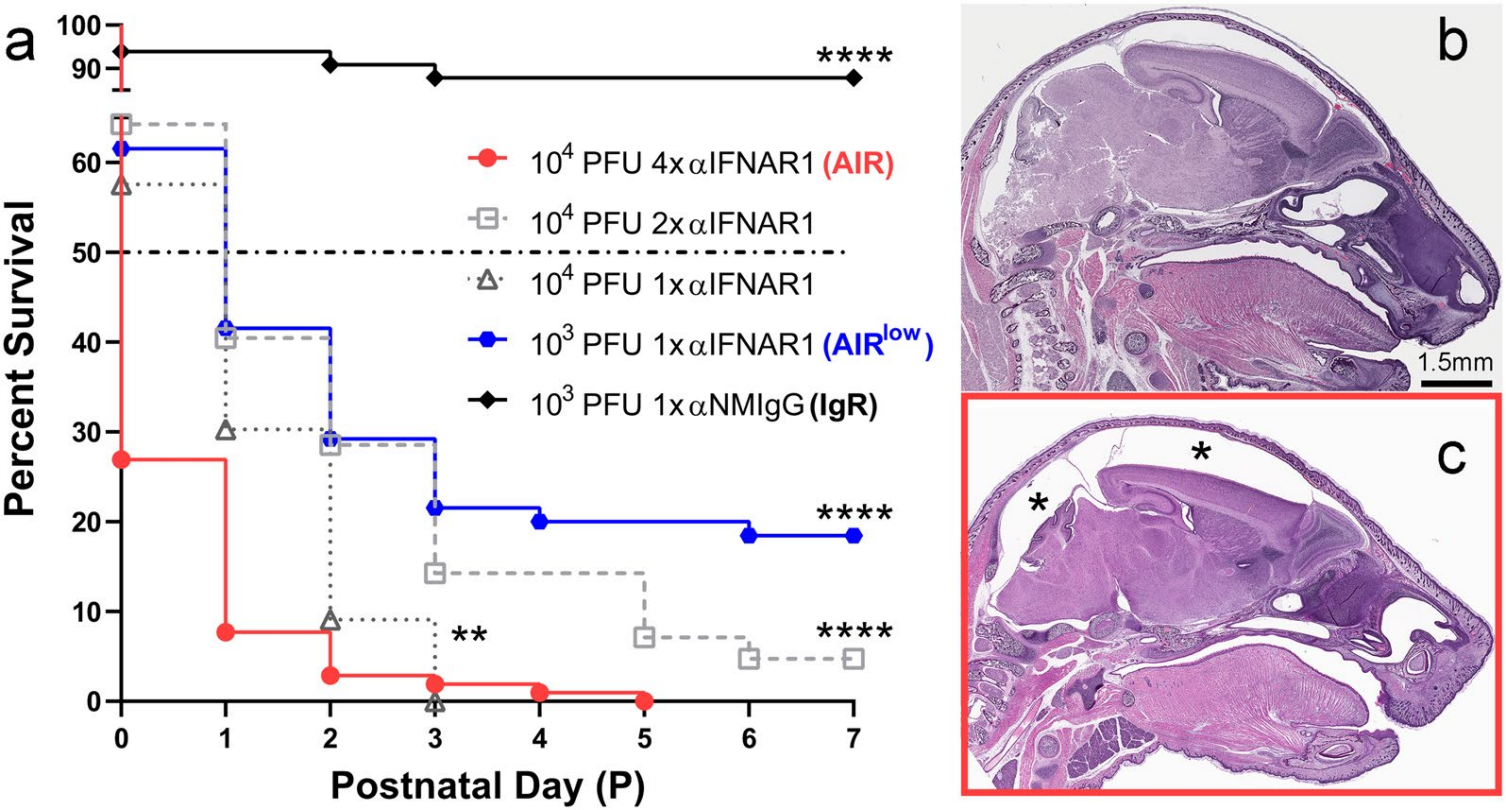
Survival of AIR pups increases with decreasing amounts of virus and anti-IFNAR antibody administered to the dam. **a** Neonates born to AIR, AIR^low^ and IgR treated, ZIKV infected pregnant mice (see Results section for different treatments) were followed for survival out to postnatal day 7 (P7) and the results are presented as Kaplan–Meier survival curves. Each curve indicates the survival of neonates from multiple dams treated with the indicated anti-IFNAR1/normal mouse IgG dose(s) and ZIKV infectious dose. The dash-dot line plotted on the Y-axis indicates median survival as determined by Kaplan–Meier. **p < 0.01, ****p < 0.0001 indicate a significant increase in survival based on a Log-rank Mantel-Cox curve comparison test. All survival curves were compared relative to the AIR (red) curve. Whole-head H&E-stained sections of an **b** IgR and **c** AIR treated neonate at P5 are shown. Notice the diminished brain volume in the (**c**, asterisks) AIR animal which corresponds to the only surviving AIR animal at P5 (**a**, red line termination). The tissue disruption in the brainstem and cerebellum of (**b**) is due to tissue processing and cutting artifact and is not associated with virus-induced pathology

*Rag1*^*−/−*^ (B6.129S7-Rag1^*tm1mom*^/J) (The Jackson Laboratory) mice were maintained on a C57BL/6 background in a breeding colony at Rocky Mountain Laboratories. Eight-to-twelve-week-old female *Rag1*^*−/−*^ mice were time-bred to *Rag1*^*−/−*^ male mice and were injected intraperitoneally (IP) with 0.5-1 mg of anti-IFNAR1 clone MAR1-5A3 to generate AIR/AIR^low^ mice or normal mouse IgG to generate IgG, *Rag1*^*−/−*^ (IgR) mice (see Results related to Fig. 1 for specific animal numbers and timing of treatment). All bred mice were infected via IP inoculation at Gestational Day 7. All experimental dams were assigned specific XZ### identifiers and their pups’ birth order was recorded by appending P#. For example, XZ395P1 indicates the first pup born to dam XZ395. Relevant to this study, all mice termed “AIR” received 10^4^ PFU of ZIKV and all those termed “AIR^low^” received 10^3^ PFU of ZIKV in 200 µl of sterile PBS. Uninfected and untreated postnatal *Rag1*^*−/−*^ mice from two different litters were used as naïve controls at both the P7 and P14 time points.

### Histopathology and immunohistochemistry

Pups from uninfected naïve dams and ZIKV-infected IgR, AIR and AIR^low^ treated pregnant dams were perfused transcardially as previously described [69] at the indicated time points. Whole pups were bisected dorsal-to-ventral along the dorsal midline to expose internal organs including brain and spinal cord and then placed in 10% neutral buffered formalin for a minimum of 7 days. Tissues were placed in cassettes and processed by a Sakura VIP-6 Tissue Tek on an automated 12 h schedule, using graded series of ETOH, xylene and PureAffin. Embedded tissues from both hemispheres were serially sectioned in the sagittal plane at 5 µm beginning at the midline bisection, mounted on slides and dried overnight at 42 °C. The 1st, 9th and 18th slides from all hemispheres were stained with hematoxylin and eosin (H&E) for histological and morphometric examination (see below) and were scanned with a Aperio AT2 slide scanner (Leica).

Sections visualized by immunofluorescence were subject to antigen retrieval with a Biocare Medical DC1674 Decloaker chamber in 0.1 M Citric acid/0.1 M Na Citrate buffer for 20 min at 120 °C/15PSI. Sections were then blocked at RT for 30 min (5% normal donkey serum, 0.01% TritonX100, 0.3 M Glycine in PBS) and primary antibodies against ZIKV NS5 (chicken, 1:1500, Aves Labs) and active Caspase 3 (rabbit, 1:250, Promega) or ionized calcium binding protein (rabbit, Iba1, 1:250, Dako) or glial fibrillary acidic protein (GFAP, rabbit, 1:250, Wako) were applied overnight at 4 °C. Species-specific secondary antibodies (goat anti-Chicken Alexa Fluor 488 and donkey anti-rabbit Alexa Fluor 594, 1:500, Thermo Fischer Scientific) were applied for 1 h at RT to label specific primaries. Hoechst (1:2000, Thermo Fischer Scientific) was applied for 10 min to label cell nuclei. Number 1.5 coverslips were applied to slides over Prolong Gold antifade mounting medium (Molecular Probes) prior to imaging. All slides were imaged using a Ziess Axio Scan Z1 (Carl Zeiss) with a 40 × plan apochromat with a numerical aperture of 0.8.

Sections visualized by dual chromogenic labeling underwent antigen retrieval performed on the Ventana Discovery ULTRA with Discovery CC1 (Roche Tissue Diagnostics) for 48 min at 90 °C. Primary antibodies against ZIKV NS2B (rabbit, 1:250, Genetex) or NeuN (rabbit, 1:100, Cell Signaling), or Calbindin-D-28 K (rabbit, 1:2250, Millipore) or Iba1 (rabbit, 1:1000, gift by John Portis (RML)) or GFAP (rabbit, 1:3000, Agilent/Dako) or transmembrane protein 119 (TMEM119, rabbit, 1:1000, Synaptic Systems) were applied for 1 h at 37 °C, except GFAP which was applied for 32 min at 37 °C. Subsequently, a species-specific secondary antibody conjugated to a hapten (NP or HQ) was applied for 8 min followed by a hapten-specific, enzyme-conjugate tertiary antibody for 8 min. Primary antibody-specific labeling was then visualized utilizing purple or yellow chromogen HRP or AP enzyme reaction detection kits (Roche Tissue Diagnostics) applied for 24 and 32 min at 37 °C respectively. For dual labeling, sections already labeled for ZIKV were treated with a heat-based denaturant step at 95 °C for 12 min to remove unbound primary and the labeling process was repeated with a different primary-secondary-enzyme conjugate combination. No-primary and single-label controls were performed for all antibodies to ensure specific staining. Following antibody labeling, hematoxylin counterstain was applied. All staining was performed using the Ventana Discovery ULTRA staining platform (Roche Tissue Diagnostics).

### Histopathologic Scoring

Tissues were evaluated blindly by a board-certified veterinary pathologist. Central (brain and spinal cord) neural tissues were evaluated for neuronal necrosis and/or degeneration, and evidence of axonal degeneration. Pathology was graded as 0 (no lesion), 1 (exceedingly rare lesions), 2 (rare, scattered lesions), 3 (moderate numbers of evenly dispersed lesions) or 4 (severe lesions).

### Morphometric brain measurements

Morphometric analyses of cortical thickness and cerebellar area were performed on all H&E-stained section sections, from both hemispheres described in the “Histopathology and Immunohistochemistry” section (see above). These were the 1st, 9th and 18th slides collected from each hemisphere, sectioning laterally from the midline bisection of each brain. Six total sections (3 from each hemisphere) were analyzed for each of 10 IgR, 3 naïve and 6 AIR^low^ pups at P7 and 10 IgR, 3 naïve and 3 AIR^low^ pups at P14. However, not all sections yielded useable measurements. Two hemispheres from P7 IgR mice and one from a P14 IgR had all three sections cut too far lateral from the midline to be comparable to other sections from the other hemispheres. All sections from these three hemispheres were discarded from the analysis. This determination was made based on the appearance of lower brain structures such as the hippocampus, thalamus and midbrain as well as the position of the third and lateral ventricles. In three other hemispheres at P7 (two IgR and one AIR^low^) and one at P14 (a naïve), the 18th section only was too far lateral using the same determinations. These three total sections were also discarded form analysis. Finally, in two hemispheres from P7 mice, (one IgR and one naïve), the 1st and 9th and 9th sections (3 total) respectively had a fold and/or cutting damage in the cerebral cortex making measurements in that region impossible. Measurements of cerebellar area were taken in those sections as that area was undamaged.

For each section, measurements were taken using annotation tools within Aperio ImageScope software (Leica) by a blinded researcher. Measurements of cortical thickness were taken by drawing an annotated straight line from the ventral aspect of cortical layer six to the surface of the brain directly above the lateral ventricle immediately anterior of the CA3 region of the hippocampus (measurement shown in relevant figure). Cortical thickness measurements were taken in µm. Measurements of cerebellar area, measured in um^2^, were taken by drawing an annotated polygon around the entirety of the cerebellar structure in each (measurement shown in relevant figure). Cerebellar external germinal layer (EGL) thickness (taken in µm) was measured for all sections from the P7 pups included in the analysis. Measurements were taken by drawing an annotated straight-line across the thickness of the EGL at the internal fold of the primary fissure (measurement shown in relevant figure). Data of cerebellar area, cortical thickness and EGL thickness were plotted as each point representing the mean of the all measurements taken from the H&E-stained sections from each hemisphere of each animal.

### Quantitative real-time PCR (qRT-PCR)

Quantitative real-time PCR (qRT-PCR) analysis of mRNA from postnatal brain was performed as previously described [68]. Primers used included *Gapdh* F (5′-TGCACCACCAACTGCTTAGC-3′), *Gapdh* R (5′-TGGATGCAGGGATGATGTTC-3′), *ZIKV* F (5′-AAGCTGAGATGGTTGGTGGA-3′), *ZIKV* R (5′-TTGAACTTTGCGGATGGTGG-3′), *Mcph1* F (5′-AAGAAGAAAAGCCAACGAGAACA-3′), *Mcph1* R (5′-CTCGGGTGCGAATGAAAAGC-3′), *Aspm* F (5′-CCGTACAGCTTGCTCCTTGT-3′), *Aspm* R (5′-GGCGTTGTCCAATATCTTTCCA-3′), *Casc5* F (5′-TCGCTGAAGTGGAAACAGAAAC-3′), *Casc5* R (5′-TATCTGAGCAAGGGTCTCTGCG-3′), *Wdr62* F (5′-GCTGACAAATGGCAAGCTG-3′), *Wdr62* R (5′-GATGGTCTTGAGGGGTTCCT-3′), *Cdk5* F (5'-GCCCTATTGGCCAAGCTACA-3'), *Cdk5* R (5'-TAAGGTCGTGAATGGTTCGGG-3'), *Cenpj* F (5'-CCTGAGTCAAGATCAACCACCA-3'), *Cenpj* R (5'-TCCAAGGCACTTTCTCGTTCA-3'), *Gfap* F (5′ CGTTTCTCCTTGTCTCGAATGAC-3′), *Gfap* R (5′- TCGCCCGTGTCTCCTTGA-3′), *Aif1* F (5′- GCCTAAGACAACCAGCGTCT-3′), *Aif1* R (5′- GACGGCAGATCCTCATCATT-3′), *Gpr84* F (5′- CTGACTGCCCCTCAAAAGAC-3′), *Gpr84* R (5′- GGAGAAGTTGGCATCTGAGC-3′), *Pcp2(L7)* F (5′- TAGACAAGGCAGGTTCACCG-3′) and *Pcp2(L7)* R (5′-CCTGGGTGTTGACCAGCATA-3′). Primers were queried with the Basic Local Alignment Search Tool (National Center for Biotechnology Information) to ensure detection of specific genes and each primer set was tested on positive and negative controls to ensure specific amplification of a single product. Data are calculated as the percent difference in threshold cycle (*C*_*T*_) value between the house-keeping gene *Gapdh* and the target gene (D*C*_*T*_ = *C*_*T*_ for *Gapdh* gene-*C*_*T*_ for target gene). Gene expression is plotted as the percent of gene expression relative to the *Gapdh* gene.

### Statistical analysis

All statistical analyses were performed using Prism 9.11 software (GraphPad). The statistical test for each experiment and the level of significance is described in the figure legends.

## Results

### Decreasing amounts of anti-IFNAR1 antibody and virus in pregnant AIR mice increases neonatal viability

ZIKV-infected, anti-IFNAR1 treated, *Rag1*^−/−^ (AIR) mice consistently transmit virus to their fetuses near the time of birth [67, 70]. However, the consequence of VTx on postnatal survival and CNS pathogenesis in this model has not been determined. To address this, we examined neonatal survival from 17 litters of AIR mice resulting in 100 offspring that were treated according to our standard model which includes a 10^4^-plaque forming unit (PFU) inoculum dose of ZIKV to mice treated with 1 mg/mouse anti-IFNAR1 on -1 and 3 dpi (days post infection) and 0.5 mg/mouse on 7 and 11 dpi (Fig. 1a, AIR, red circles). Most of the resulting pups from these pregnancies died immediately following, or within several hours of birth. Only one animal lived out to P5 (termination of red curve). This animal had an enlarged space between the surface of the brain and the skull (Fig. 1c, asterisk) relative to time-matched controls (Fig. 1b) suggesting a microcephaly-like phenotype. Other pups that survived to P3 and P4 respectively also tended to have smaller brain volumes (not shown), but to a lesser extent than was observed in the P5 animal. However, because none of these pups survived out to P7, which is analogous to full term in human CNS development, this treatment regime was not ideal for modeling postnatal ZIKV-associated CNS pathogenesis.

Next, we examined neonatal survival in conditions of decreasing amounts of anti-IFNAR1 treatment (Fig. 1a). From 8 separate dams treated with 1 mg/mouse anti-IFANR1 on -1 and 3 dpi and infected with 10^4^ ZIKV, 42 offspring were born. These pups had an increase in median age of survival from P0 to P1 (Fig. 1, light gray squares) with significantly more pups surviving longer than in the initial AIR protocol group with 4.8% surviving out to P7. Further decreasing the dose of anti-IFNAR1 administered by half by only administering anti-IFNAR antibody on -1 dpi (Fig. 1a, dark gray triangles) also resulting in increased survival relative to the AIR group but did not improve neonatal survival at P7 suggesting anti-IFNAR1 antibody treatment was not the only factor limiting survival. Thus, we decreased the inoculum dose of ZIKV from 10^4^ to 10^3^ PFU/mouse in 9 AIR dams, along with only administering anti-IFNAR1 antibody on -1 dpi. The resulting 65 offspring treated had the same increase in median survival (P1), but also had a significant increase in the number of neonates that survived out to P7 (18.5%) (Fig. 1a, AIR^low^, blue hexagons). The 12 neonatal animals from this group that survived out to P7, which will be referred to as AIR^low^ pups from here on, became the focus of further experiments to examine CNS pathogenesis as the result of ZIKV VTx. We also examined neonate survival from seven control litters of 10^3^ PFU ZIKV-infected Rag1^−/−^ dams treated with 1 mg/mouse normal mouse IgG (Fig. 1a, IgR, black diamonds) on -1 dpi. Of the resulting 52 offspring, 87.9% survived to P7. These mice along with untreated and uninfected naïve mice from two separate litters were used as controls for ZIKV VTx and CNS pathology.

### Neonates from AIR^low^ pregnancies that survived to P7 have CNS ZIKV infection

**Fig. 2:**
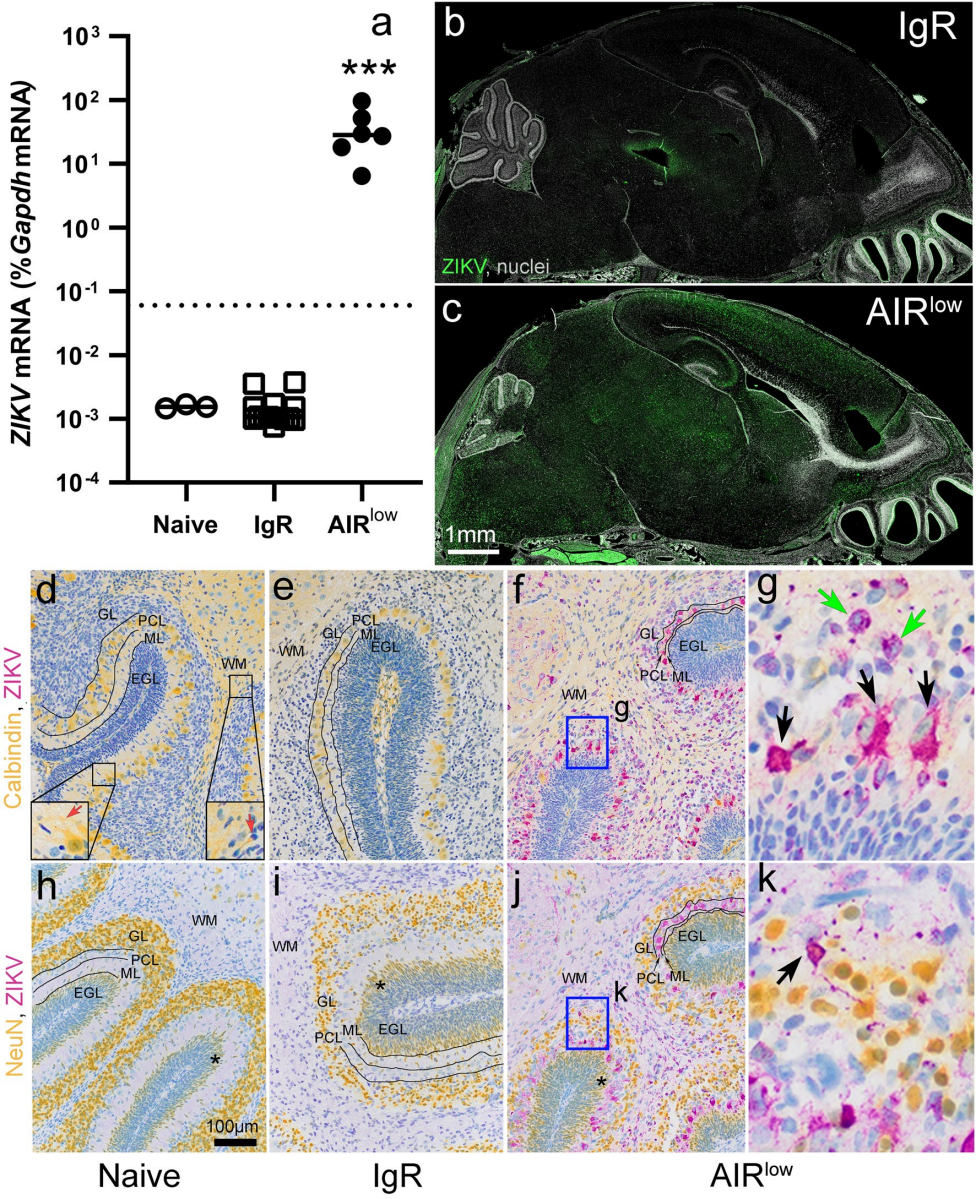
ZIKV broadly infect neurons within the CNS of P7 AIR^low^ neonates. P7 neonatal whole brain from naïve, IgR and AIR^low^ pups was evaluated for (**a**) *ZIKV* RNA by qRT-PCR with virus-specific primers. Each symbol indicates an individual brain. The mean of the individually plotted data points for each group is represented by the horizontal black bars. The dotted line in (**a**) indicated the limit of detection for the assay. Data was analyzed by a One-way ANOVA (F_2, 20_ = 12.56, *p* = 0.0003) with a Tukey Multiple Comparisons Test to determine significance between groups (***). Whole brain sections from **b** IgR and **c** AIR^low^ neonates were immunohistochemically labeled for ZIKV NS5 antigen (green) and cell nuclei (grey, Hoechst) and visualized via fluorescence microscopy. Scale bar in (**c**) applies to (**b**). Cerebellum from (**d**, **h**) naïve, (**e**, **i**) IgR and (**f**, **j**) AIR^low^ mice were dual chromogenic labeled for (**d**–**g**) Calbindin (yellow) and ZIKV NS2B (purple) or (**h**–**k**) NeuN (yellow) and ZIKV NS2B (purple) to demonstrate cellular organization and neuronal infection. The normal position and shape of the granular layer (GL), Purkinje cell layer (PCL), molecular layer (ML) and external germinal layer (EGL) are shown in a naïve mouse cerebellum labeled with (**d**) Calbindin and (**h**) NeuN at the P7 time point. Calbindin clearly labels Purkinje cell bodies in PCL (**d**, lower left inset) and dendrites in the ML (**d**, lower left inset, red arrow) and neurons associated with white matter (WM, d, lower right inset, red arrow) while NeuN labels neurons in the granular layer and maturing granular neurons as they emerge from the EGL to populate the GL. The black lines in (**e**, **i**) highlight the normal-appearing PCL and ML in an IgR mouse. These structures appear similar to those in the naïve mouse (**d**, **h**, PCL and ML). In contrast, the black lines in (**f**, **j**) highlight the diminished PCL and ML in an AIR^low^ mouse with the distance between each line having decreased. Likewise, notice the disorganization and sparsity of cells in the GL of IgR (**i**) and AIR^low^ (**j**) mice, relative to the ordered GL in the naïve mouse (**h**). The asterisks in (**h**–**j**) highlight the maturing granular neurons that expression NeuN as they emerge from the EGL to populate the GL in a naïve, IgR and AIR^low^ mouse respectively. The blue boxes in (**f**) and (**j**) corresponds to the higher magnification images in (**g**) and (**k**) respectively. The black arrows in (**g**) and (**k**) demonstrate ZIKV and neuronal marker dual-labeled infected neurons, which result in red-colored cells. In contrast, cerebellar neurons that did not label with Calbindin, but are ZIKV positive are labeled purple (green arrows in **g**). The scale bar in (**d**) also applies to (**e**, **f**, **h** and **i**)

A primary goal of this study was to examine CNS pathogenesis in neonatal mice as a result of ZIKV VTx at a time point in development analogous to full-term CNS development in humans. Thus, we assayed the surviving neonates from all experimental groups (Fig. 1a) for the presence of ZIKV in the CNS at P7 by either qRT-PCR or immunohistochemistry (IHC). RNA analysis of six surviving AIR^low^ neonates showed substantial amounts of ZIKV RNA in their brain while in naïve and IgR controls viral RNA was undetectable (Fig. 2a). The other six neonates were analyzed by IHC and also had substantial viral antigen in the CNS (Fig. 2c). IgR (Fig. 2b) and naïve controls (not shown) had no detectable CNS viral antigen. Further IHC analysis determined that ZIKV infection was specific to neurons. Chromogenic IHC for ZIKV resulted in single labeled, purple-stained cells with neuronal morphology in the cerebellum (purple, Fig. 2g, green arrows) and colabeling with Calbindin (yellow) demonstrated red, double-positive Purkinje cells (Fig. 2g, black arrows). Likewise, chromogenic labeling for ZIKV (purple) and NeuN (yellow, Fig. 2h-k) demonstrated consistently double-positive cells neurons throughout multiple brain structures in AIR^low^ pups, including cerebellum (red, Fig. 2k, black arrows), cortex, hippocampus, thalamus, brain stem and spinal cord (other regions not shown). No ZIKV antigen was observed in naïve or IgR mice (Fig. 2d, e, h and k, other brain regions not shown). In the cerebellum of naïve P7 mice, Calbindin labeled primarily Purkinje neurons in the Purkinje cell layer (PCL, Fig. 2d lower left inset) and their dendrites in the molecular layer (ML, Fig. 2d lower left inset red arrow), or neurons associated with developing white matter (WM) tracks (Fig. 2d, lower right inset and red arrow) while NeuN (Fig. 2h-j) labeled mature NeuN positive neurons in the granular layer (GL) and not Purkinje cells. Dual chromogenic IHC for ZIKV and markers of glial cells including glial fibrillary acidic protein (GFAP), ionized calcium biding adaptor 1 (Iba1) and transmembrane protein 119 (TMEM119) demonstrated no dual-positive cells suggesting glial cells were largely uninfected (data not shown).

### VTx of ZIKV into the CNS of AIR^low^ pups results in CNS structural abnormalities

**Fig. 3:**
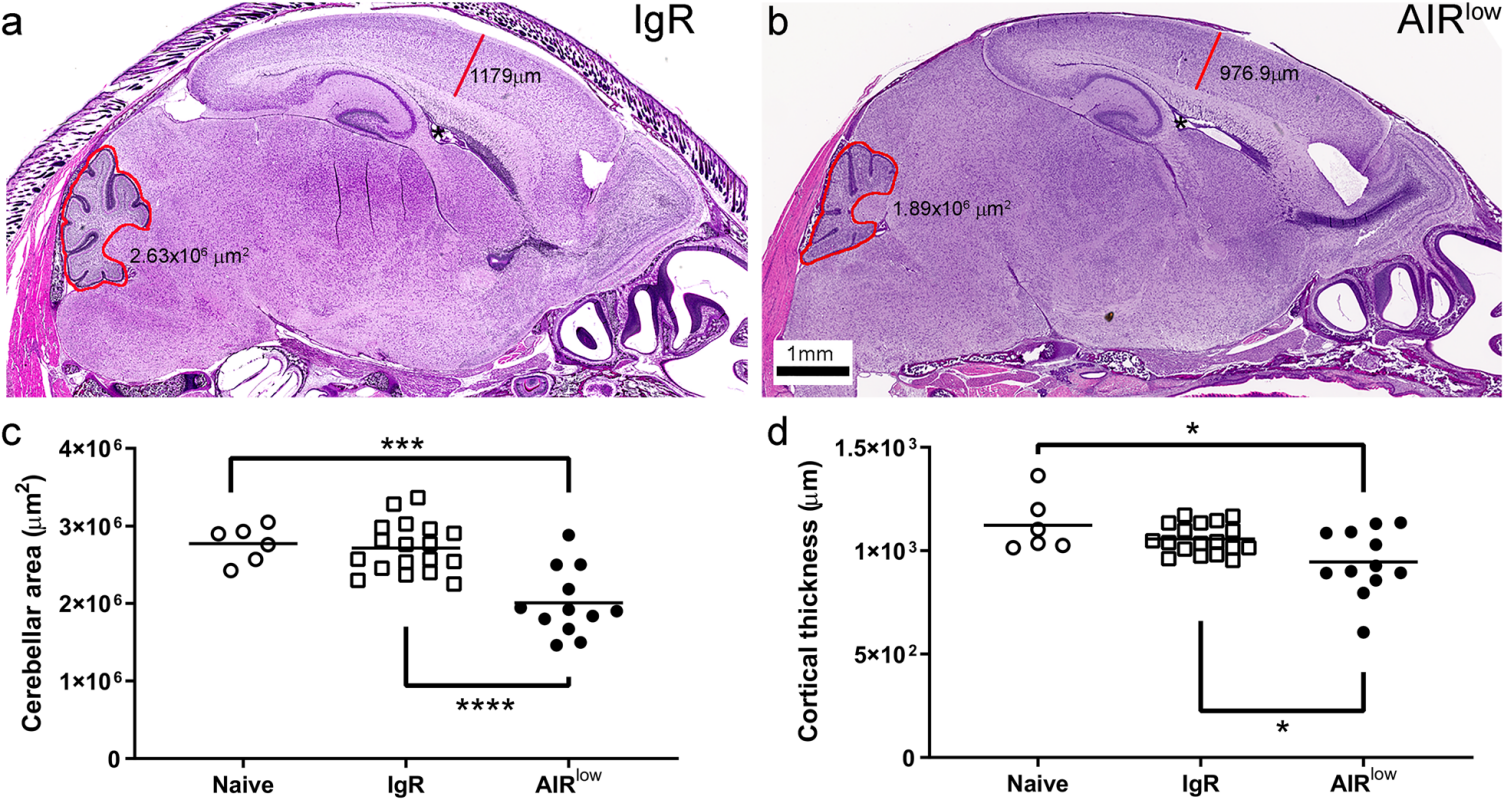
ZIKV infection in the CNS of P7 AIR^low^ neonates results in CNS structural abnormalities. H&E-stained whole brain sections from **a** IgR and **b** AIR^low^ mice are shown as representative examples of morphometric measurements taken of cortical thickness (red line in **a**, **b**) and cerebellar area (red polygon in **a**, **b**). Actual recorded values for each section are shown. The scale bar in (**b**) applies to (**a**). Morphometric measurements from each hemisphere of 3 naïve, 10 IgR and 6 AIR^low^ are shown for (**c**) cerebellar area and (**d**) cortical thickness. Each symbol represents the mean measurement from all sections for each hemisphere of each animal. The mean of the individually plotted data points for each group is represented by the horizontal black bars. A One-way ANOVA was used to compare cerebellar area (**c**, F_2, 33_ = 16.62, *p* < 0.0001) and cortical thickness (**d**, F_2, 33_ = 5.54, *p* = 0.0084) from samples from each group and a Tukey Multiple Comparisons Test was used to determine significance between groups. *p < 0.05, ***p < 0.001, ****p < 0.0001 indicate significance between groups

ZIKV infection in the CNS of fetal mice and humans can cause structural abnormalities including cortical thinning and cerebellar hypoplasia [63]. To determine if CNS infection of AIR^low^ pups caused similar abnormalities, morphometric analysis of cortical thickness and cerebellar area were performed (Fig. 3). Measurements of cerebellar area from each hemisphere of six AIR^low^ mice were significantly smaller than those from nine IgR and three naïve controls (Fig. 3c). Likewise, the thickness of the cortex directly above the lateral ventricle directly rostral of the CA3 region of the hippocampus (Fig. 3a, b, asterisks) was less in AIR^low^ mice than controls (Fig. 3d).

### P7 AIR^low^ mice do not exhibit genetic developmental deficiencies associated with microcephaly

**Fig. 4:**
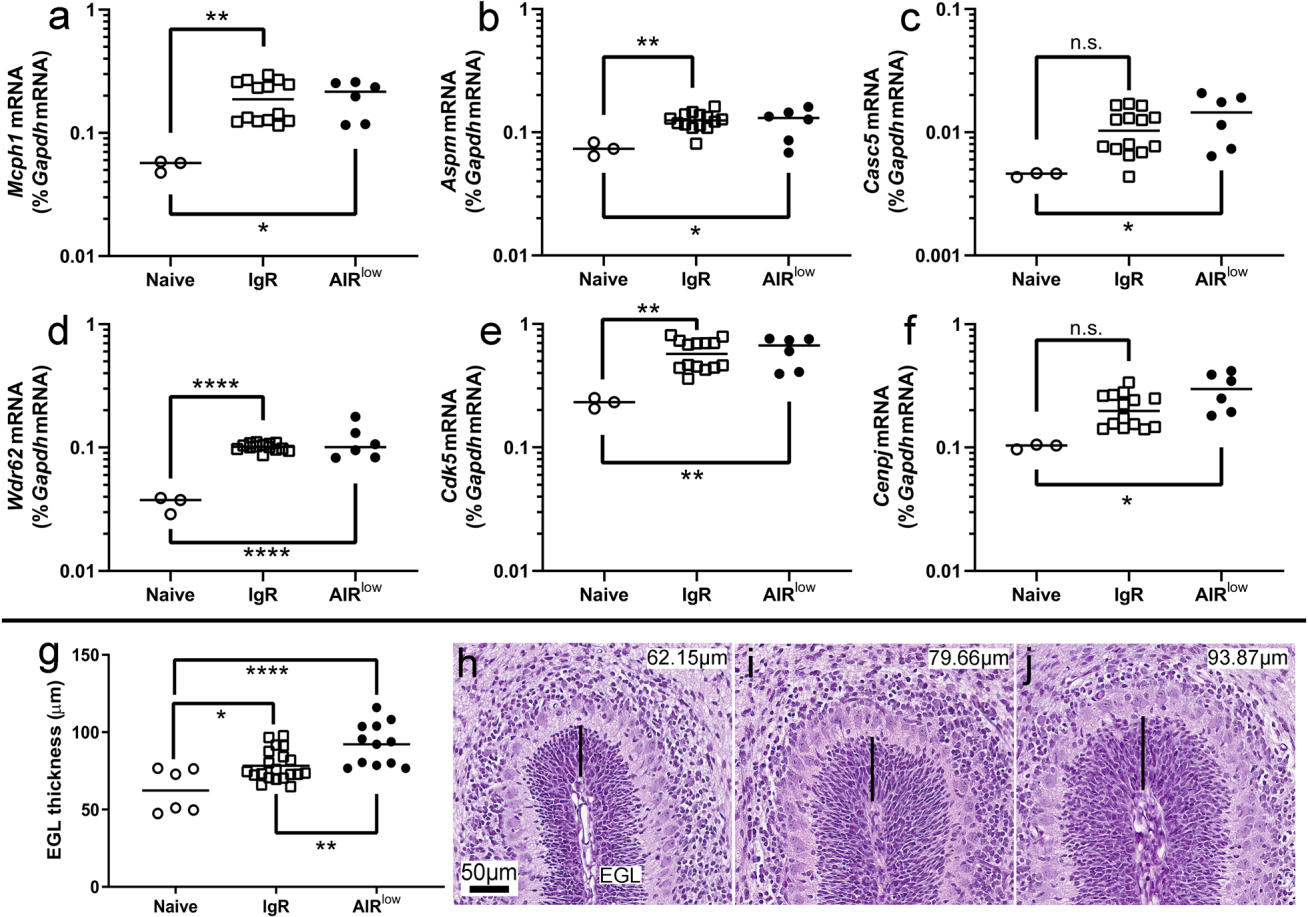
mRNA expression of microcephaly-related genes is moderately increased and cerebellar progenitor development is altered in AIR^low^ mice. P7 neonatal whole brain from naïve, IgR and AIR^low^ pups was evaluated for RNA expression of neurodevelopmental genes including **a** *Mcph1*, **b** *Aspm*, **c** *CASC5*, **d** *WDR62*, **e** *CDK5*, and **f** *Cenpj* by qRT-PCR with specific primers. Each symbol indicates an individual brain. The mean of the individually plotted data points for each group is represented by the horizontal black bars. A One-way ANOVA was used to compare RNA expression of **a** *Mcph1* (F_2, 20_ = 5.98, *p* = 0.0092), **b** *Aspm* (F_2, 20_ = 5.66, *p* = 0.0113), **c** *CASC5* (F_2, 20_ = 3.99, *p* = 0.0349), **d** *WDR62* (F_2, 20_ = 17.79, *p* < 0.0001), **e** *CDK5* (F_2, 20_ = 7.21, *p* = 0.0044), and **f** *Cenpj* (F_2, 20_ = 7.33, *p* = 0.0041) from samples from each group and a Tukey Multiple Comparisons Test was used to determine significance between groups. *p < 0.05, **p < 0.01, ****p < 0.0001, indicate significance between groups. n.s. = not significant. The (**g**) thickness of the EGL at the internal fold of the primary cerebellar fissure was measured in (**h**) 3 naïve, (**i**) 9 IgR and (**j**) 6 AIR^low^ pups at P7. In (**g**), each symbol represents the mean measurement from all sections for each hemisphere of each animal. The mean of the individually plotted data points for each group is represented by the horizontal black bars. A One-way ANOVA was used to compare EGL thickness (**g**, F_2, 35_ = 13.02, *p* < 0.0001) from samples from each group with a Tukey Multiple Comparisons Test to determine significance between groups. *p < 0.05, **p < 0.01, ****p < 0.0001 indicate significance between groups. In (**h**–**j**) the black line indicates the measured thickness of the EGL and actual recorded measurements are shown in the top right. The scale bar in (**h**) applies also to (**i** and **j**)

ZIKV infection in the fetal CNS can decrease the expression of key developmental genes linked to congenital microcephaly which promote neural progenitor division and normal brain development [43, 66]. qRT-PCR analysis of six gene transcripts associated with microcephaly was conducted on whole-brain RNA from P7 AIR^low^, naïve and IgR pups (Fig. 4a-f). Contrary to published work in mice with vertically transmitted ZIKV [27, 43], AIR^low^ mice (filled black circles) with active CNS infection expressed higher mRNA levels of all six neurodevelopmental genes relative to uninfected naïve controls. IgR controls (open squares), which did not have active CNS infection at P7, also trended toward higher neurodevelopmental gene mRNA expression with only *Casc5* and *Cenpj* not reaching statistical significance. mRNA expression in IgR mice was consistently intermediate to naïve and AIR^low^ expression suggesting active ZIKV infection in the CNS induced higher neurodevelopmental gene expression.

### P7 AIR^low^ mice have altered cerebellar progenitor development

It is possible that ZIKV-induced increased postnatal neurodevelopmental gene expression could reflect alterations in neural progenitor development. To try and better understand this finding, the thickness of the progenitor cell-containing external germinal layer (EGL) and the distribution of mature neurons in the highly organized cerebellum [23] was examined in all three experimental groups. During neonatal development, the thickness of the EGL is associated with the rates of progenitor maturation, migration away from the EGL and progenitor self-renewal within the EGL [6]. Thus, changes in EGL thickness can indicate alterations in progenitor development. Naïve control pups (Fig. 4h) demonstrated a time point-specific normally sized EGL where proliferating granular cell progenitor neurons arise before migrating to the GL as mature neurons. In contrast, the EGL in IgR (Fig. 4i) and AIR^low^ (Fig. 4j) mice was enlarged (Fig. 4g), relative to naïve pups suggesting increased progenitor proliferation correlating with higher neurodevelopmental gene expression. In further support of this idea, more cells expressing the mature neuronal marker NeuN were observed on the border of the EGL in IgR (Fig. 2i, asterisk) and AIR^low^ (Fig. 2j, asterisk) mice as compared to naïve mice (Fig. 2h, asterisk). Possibly, more progenitor cells were maturing and beginning their migration into the GL for IgR and AIR^low^ mice compared to naïve controls.

Naïve mice had a clearly defined PCL, which contains Calbindin-positive Purkinje cell bodies (Fig. 2d, lower left inset) and ML, which contains Calbindin-positive dendrites of Purkinje cells (Fig. 2d, lower left inset, red arrow), but can also be identified by its neuroanatomical position (Fig. 2d, black lines identify the two layers). They also have an established GL populated by mature neurons as indicated by NeuN IHC (Fig. 2h). In IgR mice, the PCL (Fig. 2e, black lines) and the ML (black lines) appeared well formed and similar in thickness to naïve mice (Fig. 2d). However, in all animals examined, the NeuN positive cells within the GL of IgR mice (Fig. 2i, GL) appeared disorganized and sparse with variable spacing between cells compared to naïve controls, in whose GL neurons were tightly packed (Fig. 2h, GL). AIR^low^ mice had active infection in the PCL which is correlated with thinning of that layer and the ML as shown by Calbindin labeling (Fig. 2g, f, black lines). Like IgR mice, AIR^low^ mice had a sparsely populated, and disorganized GL as shown by NeuN labeling (Fig. 2j) but to an even greater extent than in IgR mice (Fig. 2i).

### VTx of ZIKV to P7 neonates results in CNS cell damage and death

**Fig. 5:**
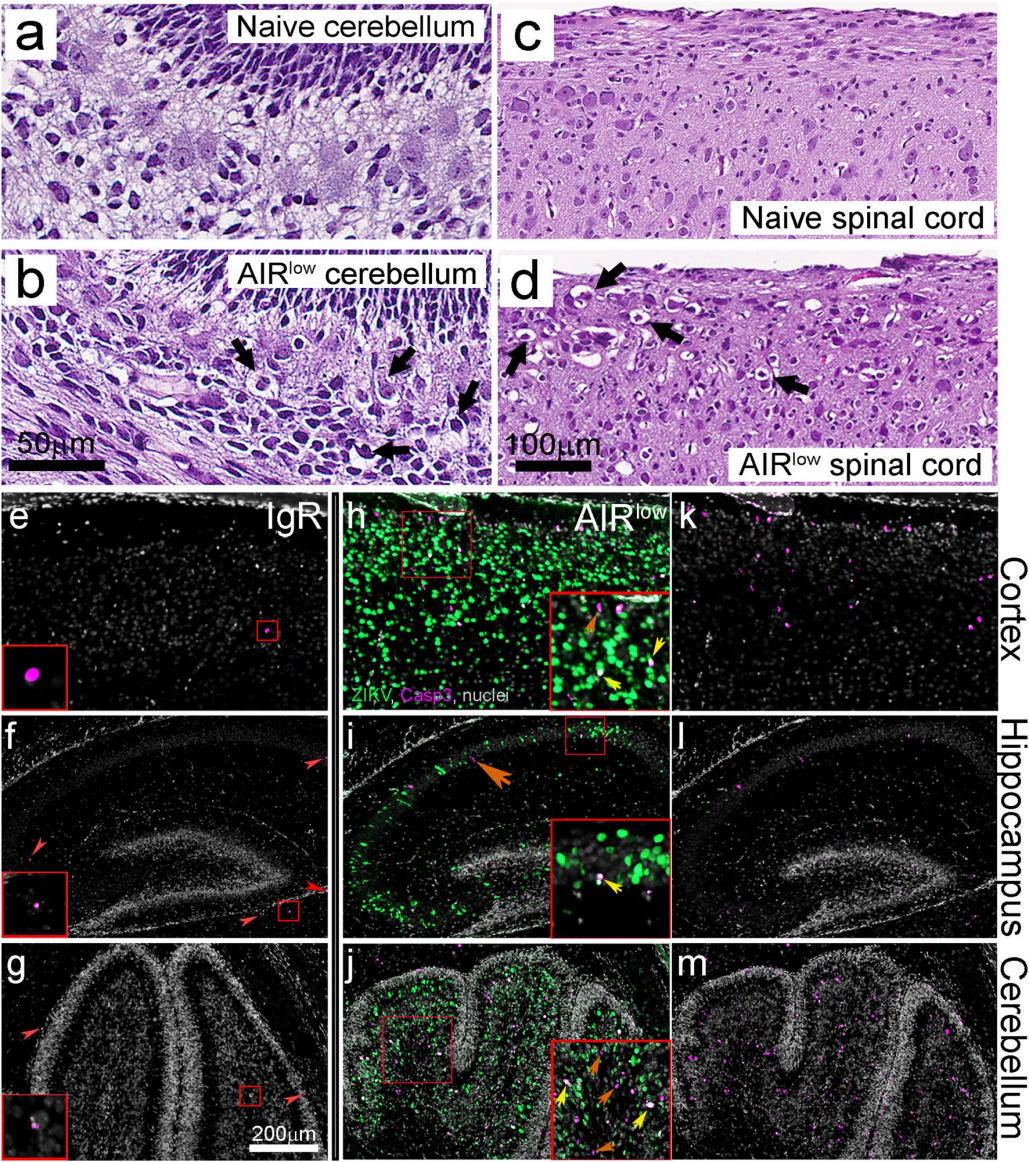
ZIKV infection increases cell death in the CNS of P7 AIR^low^ neonates primarily in the cerebellum, cortex and spinal cord. Representative H&E-stained sections from P7 (**a**, **c**) naïve and (**b**, **d**) AIR^low^ cerebellum and spinal cord respectively. Degenerating cerebellar neurons (**b**, black arrows) and axonal degeneration (**d**, black arrows) were observed in AIR^low^ mice. The scale bar in (**b**) applies to (**a**) and the bar in (**d**) applies to (**c**). Representative immunofluorescence labeled CNS sections from P7 (**e**–**g**) IgR and (**h**–**j**) AIR^low^ pups demonstrate ZIKV NS5 (green) and active-Caspase 3 (magenta) positive cells in cortex, hippocampus and cerebellum. Nuclei are shown in grey. Images of active-Caspase 3 and nuclei staining only in AIR^low^ mice are shown in (**k**–**m**). Red arrows and box insets in (**e**–**g**) indicate baseline active-Caspase 3 labeling in control animals. Red boxes in (**h**–**j**) correspond to insets in each specific brain region. Yellow arrows indicate ZIKV/active-Caspase 3 dual positive cells while orange arrows indicate active-Caspase 3 only cells. The scale bar in (**g**) applies to (**e**–**m**)

ZIKV induces cell death when directly injected into fetal and neonatal mice [27, 52] which is a possible mechanism contributing to CZS in humans. Thus, we examined the CNS of surviving P7 AIR^low^ and control neonates for indications of cellular death as a result of vertically transmitted ZIKV infection. H&E-stained sections from all six AIR^low^ mice from 3 different litters demonstrated minimal to moderate neuronal degeneration throughout the CNS (Table 1). Degenerating Purkinje and granular layer neurons of the cerebellum (Fig. 5b, arrows) and motor neurons of the spinal cord undergoing axonal degeneration (Fig. 5d, arrows) were among the most commonly observed pathologies. All naïve control sections were normal and contained no pathology (Fig. 5a and c). Two of 10 IgR control samples showed minimal-to-mild neuronal degeneration and only in pups from a single litter (Additional file 1: Fig. S1). Degeneration in these two samples consisted of scattered cells primarily in the GL of the cerebellum and (Additional file 1: Fig. S1a and b) rare Purkinje neurons (Additional file 1: Fig. S1c). All other IgR samples were developmentally normal without obvious neuropathology. These data demonstrate neuronal degeneration occurs in the CNS of all AIR^low^ mice at P7 across multiple litters but is rare and less severe in controls.

**Table 1:** Scoring of CNS pathology in P7 naïve, IgR and AIR^low^ mice

Pup ID	Treatment	Brain Neuronal degeneration, cell death	Spinal cord Neuronal degeneration, cell death	Spinal cord axonal degeneration
XZ394P1	naïve	0	0	0
XZ394P2	naïve	0	0	0
XZ394P3	naïve	0	0	0
XZ3601P5	IgR	0	0	0
XZ3601P6	IgR	0	0	0
XZ3601P7	IgR	0	0	0
XZ3611P4	IgR	0	0	0
XZ3611P5	IgR	0	0	0
XZ3611P6	IgR	0	0	0
XZ383P5	IgR	0	0	0
XZ383P6	IgR	0	0	0
XZ384P4	IgR	1	0	0
XZ384P5	IgR	2	0	1
XZ357P4	AIR^low^	2	2	1
XZ357P5	AIR^low^	2	2	1
XZ357P6	AIR^low^	2	3 (ganglion)	1
XZ386P3	AIR^low^	1	0	1
XZ387P2	AIR^low^	2	1	2
XZ387P3	AIR^low^	1	0	0

Scoring of brain tissue pathology in P7 mice. H&E-stained tissue samples were evaluated in detail and the following scoring system was applied: 0 = no lesions; 1 (exceedingly rare lesions), 2 (rare, scattered lesions), 3 (moderate numbers of evenly dispersed lesions) or 4 (severe lesions)

To determine the coincidence of infection and neuronal cell death, IHC labeling for ZIKV antigen (green) and active-caspase3 (magenta) was performed on all three experimental groups (Fig. 5e–m, naïve not show). Sections from an IgR control in the cortex, hippocampus and cerebellum demonstrated a base-line amount of caspase3-dependent cell death at P7 in the absence of CNS ZIKV infection (Fig. 5e–g red arrow heads and red box insets, Additional file 1: Fig. S1d-h). In the brains of AIR^low^ pups, ZIKV antigen was readily detected in all three brain areas (Fig. 5h–m) accompanied with increased numbers of caspase3 positive cells relative to naïve and IgR controls. Higher magnification insets in these regions (Fig. 5n-p, red squares enlarged in h-j) demonstrated ZIKV/active capase3 double positive cells (yellow arrows) suggesting infected cells were undergoing apoptosis. Additionally, caspase3 single-positive cells were also frequently found in areas of infection (Fig. 5i, n, p, orange arrows) suggesting a bystander cell-death mechanism.

### ZIKV infection in the CNS of AIR^low^ pups results in activated glial cells

**Fig. 6:**
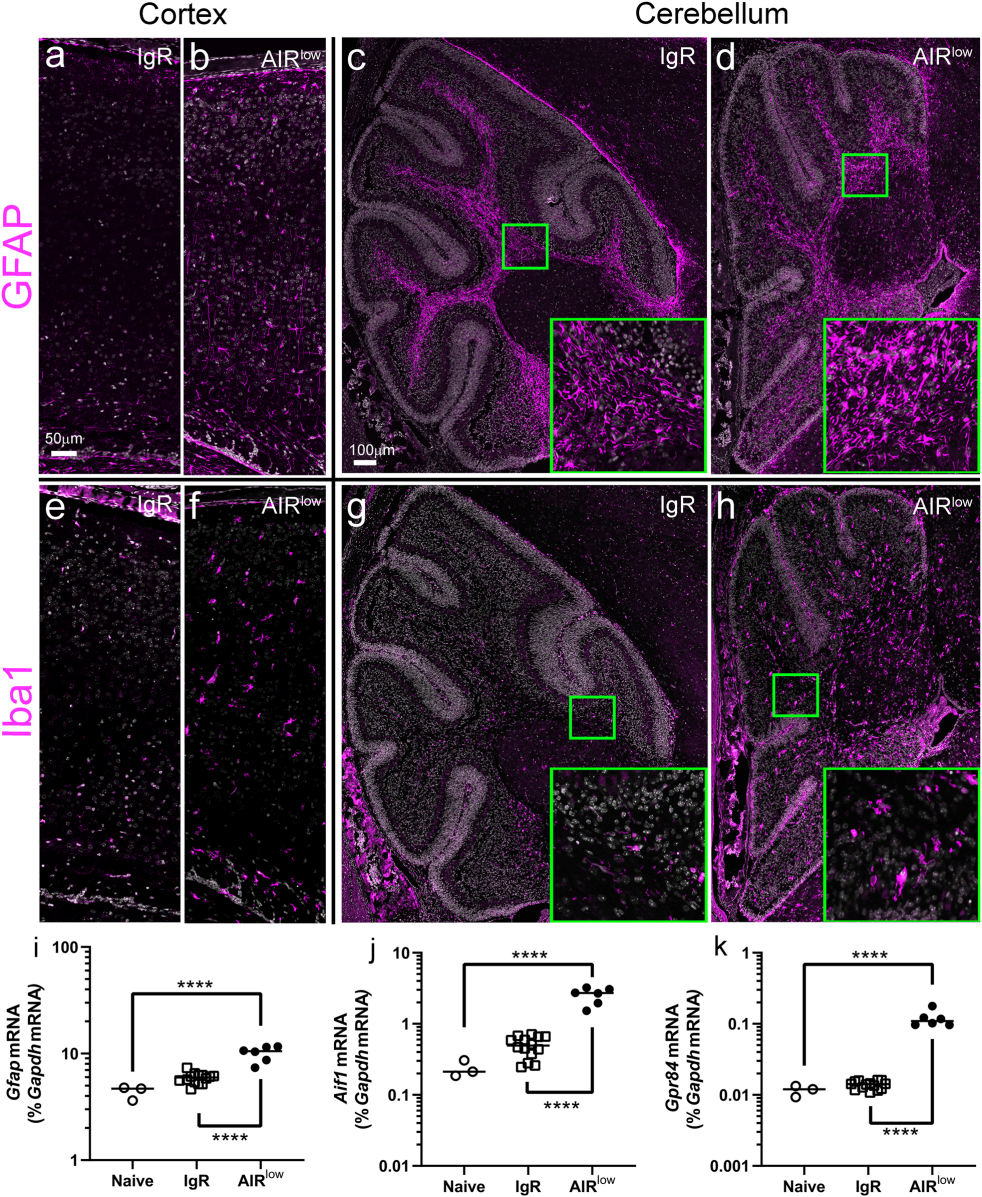
ZIKV infection induces glial activation in the CNS of P7 AIR^low^ neonates. Representative sections from P7 (**a**, **c**, **e** and **g**) IgR and (**b**, **d**, **f** and **h**) AIR^low^ mice demonstrating (**a**–**d**) GFAP and (**e**–**h**) Iba1 immunofluorescence labeling in the cortex and cerebellum. Specific labeling from each antibody is shown in magenta and cell nuclei (Hoechst) are shown in grey for counterstain. Green boxes in (**g**) and (**h**) correspond to high magnification insets to demonstration cellular morphology. P7 neonatal whole brain from naïve, IgR and AIR^low^ pups was evaluated for RNA expression of glial-specific genes including (**i**) *Gfap*, (**j**) *Aif1* (Iba1) and (**k**) *Gpr84* by qRT-PCR with specific primers. Each symbol indicates an individual brain. The mean of the individually plotted data points for each group is represented by the horizontal black bars. A One-way ANOVA was used to compare RNA expression of (**i**) *Gfap* (F_2, 20_ = 44.50, *p* < 0.0001), (**j**) *Aif1* (F_2, 20_ = 76.88, *p* < 0.0001) and (**k**) *Gpr84* (F_2, 20_ = 103.2, *p* < 0.0001) from samples from each group and a Tukey Multiple Comparisons Test was used to determine significance between groups. ****p < 0.0001, indicates significance between groups

ZIKV infection can induce glial cell activation [53] which can cause neuronal death [17, 44]. To examine the state of glial cells in the CNS of naïve, IgR and AIR^low^ mice, we performed cortical and cerebellar IHC for GFAP, a marker on astrocytes and Iba1, a marker on microglia and infiltrating myeloid cells (Fig. 6). Each marker is known to have increased expression following glial activation [39]. Sections from naïve (not shown) and IgR pups demonstrated base-line levels of GFAP (Fig. 6a, c) and Iba1 (Fig. 6e, g) labeling in the absence of ZIKV infection. In contrast, AIR^low^ pups had increased expression of both markers throughout the brain especially in the cerebral cortex (Fig. 6b, f) and cerebellum (Fig. 6d, h insets v. insets in c and g). Corresponding to increased Iba1 expression were morphological changes in Iba1-positive cells (Fig. 6 insets in g and h) with those found in AIR^low^ mice having thicker, shortened processes indicative of activated microglia. Interestingly, there was no evidence of perivascular cuffing or peripheral myeloid cell infiltrate in AIR^low^ mice suggesting that the activated cells were not infiltrating cells, but rather microglia (Additional file 2: Fig. S2). To confirm and expand on these findings, we performed qRT-PCR on whole brains from naïve, IgR and AIR^low^ pups assaying for *Gfap*, Iba1 (*Aif1*) and g-protein 84 (*Gpr84*) mRNA expression (Fig. 6i-k). Gpr84 is an orphan receptor whose expression is induced specifically on microglia in response to immune activation [28]. mRNA expression of each of these targets was significantly increased in AIR^low^ mice relative to controls. Thus, vertically transmitted ZIKV infection in the postnatal brain results in glial cell activation.

### Prolonged ZIKV infection in the CNS of AIR^low^ pups results in clinical disease

**Fig. 7:**
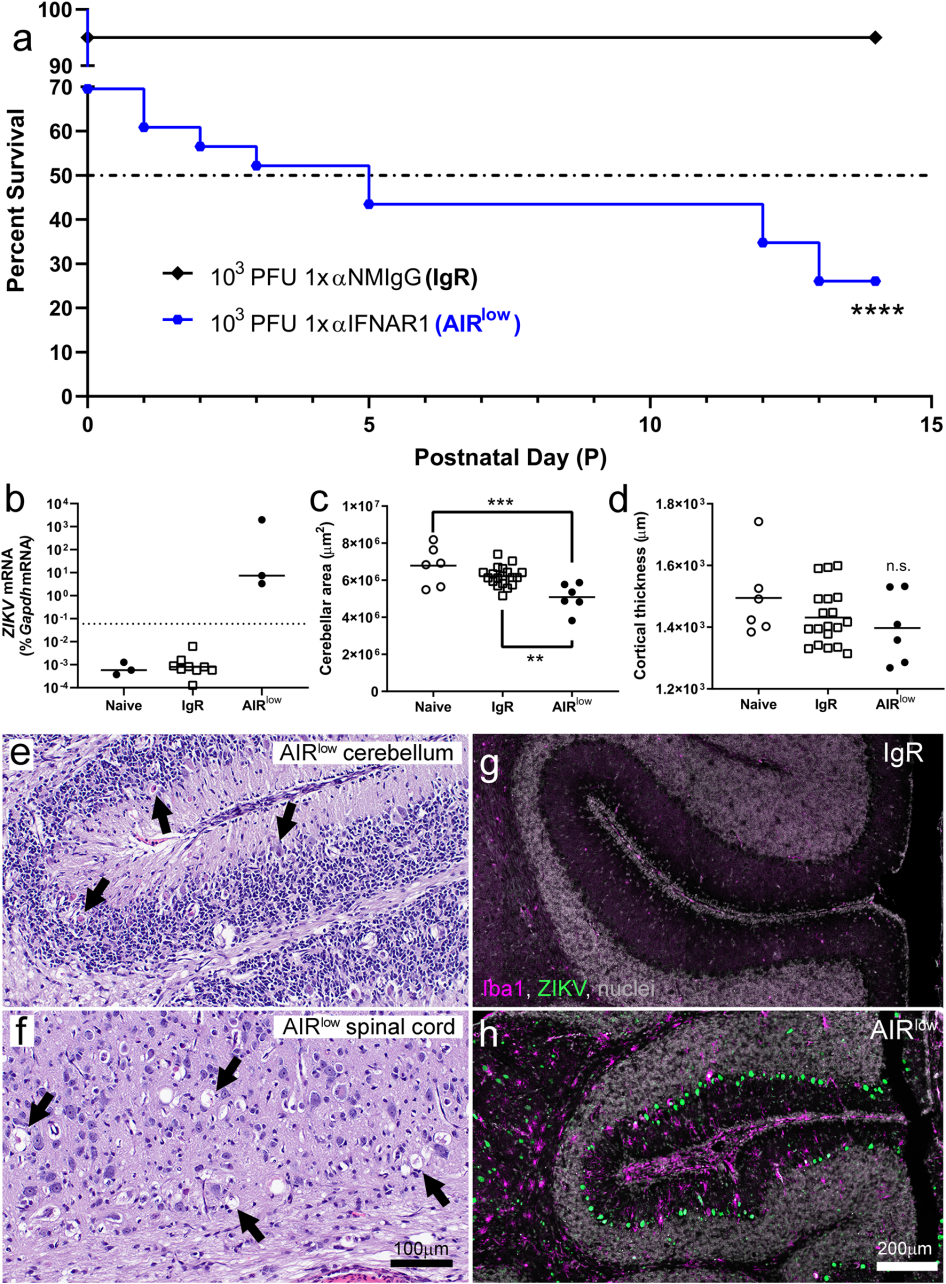
Prolonged ZIKV infection in AIR^low^ mice results in sustained CNS structural abnormalities and glial activation leading to eventual clinical neurologic disease. **a** Neonates born to AIR^low^ (blue line) and IgR (black line) treated, ZIKV infected, pregnant mice (see Results for specific mouse numbers) were followed for survival out to P14 and the results are presented as Kaplan–Meier survival curves. Each curve indicates the survival of neonates from multiple dams treated with the indicated anti-IFNAR1/normal mouse IgG dose(s) and ZIKV infectious dose. The dash-dot line plotted on the Y-axis indicates median survival as determined by Kaplan–Meier. ****p < 0.0001 indicate a significant decrease in survival based on a Log-rank Mantel-Cox curve comparison test where the AIR^low^ curve was compared to the IgR. **b** P14 naïve, IgR and AIR^low^ pups were evaluated for *ZIKV* RNA by qRT-PCR with virus-specific primers. The dotted line in (**b**) indicates the limit of detection for the assay. Morphometric measurements from each hemisphere of 3 naïve, 10 IgR and 3 AIR^low^ mice that survived to P14 are shown for (**c**) cerebellar area and (**d**) cortical thickness. Each symbol represents the mean of 3 measured sections from each hemisphere of each animal. The mean of the individually plotted data points for each group is represented by the horizontal black bars. A One-way ANOVA was used to compare cerebellar area (**c**, F_2, 28_ = 9.64, *p* = 0.0007) and cortical thickness (**d**, F_2, 28_ = 1.384, *p* = 0.2673) from samples from each group and a Tukey Multiple Comparisons Test was used to determine significance between groups. **p < 0.01, ***p < 0.001, indicate significance between groups. n.s. = not significant. Representative H&E-stained sections from P14 AIR^low^ (**e**) cerebellum and (**f**) spinal cord demonstrate degenerating cerebellar neurons (**e**, black arrows) and axonal degeneration (**f**, black arrows). The scale bar in (**f**) applies to (**e**). Representative cerebellar sections from (**g**) IgR and (**h**) AIR^low^ pups demonstrating ZIKV NS5 (green) and Iba1 (magenta) immunofluorescence labeling. The scale bar in (**h**) applies to (**g**)

The pathologic consequence of prolonged ZIKV infection in the CNS in the AIR^low^ model has not been determined. Therefore, two additional AIR^low^ and three IgR litters were treated, infected and followed for survival to P14 (Fig. 7a). Twenty neonates resulted from IgR litters and 23 from AIR^low^ litters. All but one of the IgR pups survived to P14 (95.0%) while only six of the AIR^low^ survived (26.1%) demonstrating significantly reduced survival in the latter (Fig. 7a). Of the six surviving pups, two presented on P14 with clinical symptoms including ataxia, head tilt, tremor and hind-limb paralysis. In the preceding two days (P12 and P13) an additional four AIR^low^ pups were found dead during morning health-checks after appearing neurologically normal the day before. Collectively these data suggest AIR^low^ mice developed sudden onset neurologic disease as early as P12.

qRT-PCR and IHC analysis of CNS tissues from AIR^low^ mice determined these mice had persistent ZIKV infection while naïve and IgR controls were negative (Fig. 7b and g vs h). Similar to P7 findings, morphometric analysis of brains from three naïve, 10 IgR and 3 AIR^low^ mice determined that cerebellar area was significantly reduced in P14 AIR^low^ pups (Fig. 7c) and cortical thickness also trended lower but did not reach significance (Fig. 7d). Transcriptional expression of genes associated with proliferation and neurodevelopment in IgR and AIR^low^ mice trended higher than naïve controls but did not reach statistical significance (Additional file 3: Fig. S3b-g).

Consistent with the development of neurologic disease in P14 AIR^low^ pups, more severe CNS pathology was observed in these animals compared to time-matched naïve or IgR controls (Table 2) or to P7 AIR^low^ (Tables 1 and 2) pups. The cerebellum of P14 AIR^low^ mice was highly affected, with degenerating Purkinje and granular neurons frequently observed (Fig. 7e) and decreased expression of the Purkinje cell specific Pcp2(L7) transcript (Additional file 3: Fig. S3a). Additionally, the spinal cord displayed significant axonal degeneration (Fig. 7f). IHC labeling of glial cells demonstrated highly activated astrocytes (not shown) and microglial (Fig. 7h) in AIR^low^ but not control pups (Fig. 7g).

**Table 2:** Scoring of CNS pathology in P14 naïve, IgR and AIR^low^ mice

Pup ID	Treatment	Brain Neuronal degeneration, cell death	Spinal cord Neuronal degeneration, cell death	Spinal cord axonal degeneration
XZ395P1	naïve	0	0	0
XZ395P2	naïve	0	0	0
XZ395P3	naïve	0	0	0
XZ388P4	IgR	0	0	0
XZ388P5	IgR	0	0	0
XZ388P6	IgR	0	0	0
XZ388P7	IgR	0	0	0
XZ389P4	IgR	0	0	0
XZ389P5	IgR	0	0	0
XZ389P6	IgR	0	0	0
XZ389P7	IgR	0	0	0
XZ390P4	IgR	0	0	0
XZ390P5	IgR	0	0	0
XZ392P1	AIR^low^	3	2	2
XZ393P1	AIR^low^	3	2	2
XZ393P2	AIR^low^	1	2	2

Scoring of brain tissue pathology in P14 mice. H&E-stained tissue samples were evaluated in detail and the following scoring system was applied: 0 = no lesions; 1 (exceedingly rare lesions), 2 (rare, scattered lesions), 3 (moderate numbers of evenly dispersed lesions) or 4 (severe lesions)

## Discussion

The original doses of anti-IFNAR1 and ZIKV in pregnant *Rag1*^*−/−*^ mice previously used for the AIR model of VTx caused almost uniform death of resulting pups prior to P7. The fatal disease in AIR pups at a comparatively fetal neurodevelopmental stage could be in part due to fetal growth restriction, similar to other published reports involving significant placental infection [32] because significant placental infection is observed with this model [70]. It is also possible that infectious loads are high in the brains of AIR neonates causing severe disease in the early postnatal period [67]. Reducing the amounts of anti-IFNAR1 antibody and more critically, virus, resulted in one fifth of the offspring surviving to P7 (Fig. 1) and in some cases, as long as P14 (Fig. 7). P7 is a critical stage to reach in rodents because it is roughly equivalent to full-term fetuses in humans where neurodevelopment is ongoing [50]. AIR^low^ mice also developed fatal neurologic disease, but not until approximately 2 weeks later (Fig. 7) when primary neurogenesis for all brain regions is nearing completion [50] corresponding to ~ 2 years old in humans. These mice also had persistent neuronal infection (Figs. 2, 7), CNS structural abnormalities (Figs. 3, 7), neuronal degeneration (Figs. 5, 7) and gliosis (Figs. 6, 7) which are known features of CZS [63]. Thus, the AIR and AIR^low^ models could provide and interesting comparison between fetal and postnatal ZIKV disease and the effect of infection on neurodevelopment at each timepoint.

Structural brain abnormalities in AIR^low^ mice were evident in both the cerebral cortex and the cerebellum (Figs. 3 and 7), although more pronounced in the latter. Cortical and cerebellar abnormalities are common and characteristic of ZIKV congenital disease [63] indicating the mechanisms causing these abnormalities could be relevant to humans. Previous work suggests that ZIKV infection of the fetal brain results in infection and depletion of progenitor cells [27, 43, 58, 71] which could contribute to the reduced brain volume observed at birth in both animal models and humans. However, these findings do not account for the post-natal effects of ZIKV. The cerebral cortex is largely populated by neurons derived from the ventricular and subventricular zone prior to birth [50, 56] which could account for the less pronounced structural abnormally in AIR^low^ mice as the cortex is mostly formed by the time of CNS infection. In contrast, cerebellar neural patterning happens more slowly, extending past birth [61] when granule neurons that are involved in motor coordination, cognition, emotion, and spatial navigation migrate from the EGL to populate the granular layer [23]. Thus, active postnatal ZIKV CNS infection may more profoundly impact cerebellar development than cortical neurodevelopment accounting for the persistently smaller cerebellar area in AIR^low^ mice. Similar results have been shown in postnatal infected rhesus macaques [45] suggesting this could be an important mechanism contributing to postnatal secondary microcephaly and autism that has been shown to manifest in previously healthy infants born to ZIKV infected mothers [37].

At P7, AIR^low^ mice (Fig. 5h-m) visually had more apoptotic cells than controls (Fig. 5e-g) in multiple areas of the brain including the cerebral cortex, but most notably the cerebellum, that was both dependent and independent of ZIKV infection in the cell. This increased cell death could be contributing to the observed brain abnormalities (Fig. 3) as ZIKV infection has been shown to induce apoptosis in neurons at multiple maturation stages [4] possibly through a mechanism that involves mitochondrial fragmentation [72] and aberrant nicotinamide adenine dinucleotide metabolism [42]. However, ZIKV has also been shown to cause non-autonomous induced cell death by promoting cellular expression of soluble neurotoxic factors [41]. Proinflammatory cytokines such as interleukin-6 (IL6) can function as neurotoxic factors [54] and are produced by activated CNS glial cells in response to ZIKV infection [15, 64]. Thus, it is possible the activated astroglia and microglia observed specifically in AIR^low^ mice (Figs. 6 and 7) may be contributing to infection-independent apoptosis and contributing to disease. Indeed, IL6 has been shown to causes cerebellar granule neuron death during development [9]. This may in part explain the reduced cerebellar area observed in AIR^low^ mice.

Our data agree with previously published work demonstrating ZIKV infection during pregnancy in mice alters normal brain development (Fig. 3) [10, 27, 58, 71]. This works expands on these studies to suggest that persistent postnatal infection more severely impacts development and causes severe disease compared to transient infection during gestation [21, 43, 52]. Surprisingly, this work also indicates that even during active CNS infection, expression of CNS mitotic transcripts can increase postnatally relative to naïve controls, and the neural progenitor-containing EGL of the cerebellum is enlarged suggestive of increased progenitor proliferation (Fig. 4). These features were observed in both IgR and AIR^low^ pups suggesting that maternal infection alone is sufficient to increase transcript expression and progenitor proliferation. However, the increase in mitotic transcripts was higher in AIR^low^ than IgR pups and the EGL was also significantly thicker in AIR^low^ pups suggesting active infection is a more potent driver of these processes. There are multiple possible mechanistic explanations for these results. First, it is possible the increased mitotic gene expression and thickness of the EGL observed in IgR and AIR^low^ at P7 mice may be a postnatal compensatory developmental mechanism to replace granule neurons impacted by ZIKV infection and virus-induced cell death. Alternatively, ZIKV infection may simply be disrupting the timing of cerebellar proliferation as has been demonstrated in some genetic knockout models where granule neuron proliferation is prolonged [25]. Additional experimentation will be required to determine the root causes of these differences. Regardless, these data indicate a potential therapeutic window of antiviral intervention in the early postnatal period that closes around P14 when neuronal generation slows in neurodevelopment [50] and mice develop fatal neurologic disease. This critical timepoint may also be important in human disease.

AIR^low^ mice develop fatal neurologic disease on or around P14 (Fig. 7). Indeed, we observed clinical features such as impaired gait, hind-limb paralysis and ataxia which are indications of both cerebellar and spinal neuronal death [2, 49]. Thus, fetal disease in AIR^low^ mice is likely in part the result of cell death within these important CNS structures. As discussed above, direct infection of neurons in the cerebellum could account for the degenerating cells observed in that structure. Likewise, infection in the spinal cord can result in axonal degeneration [33, 59] causing motor neuron loss [11, 14]. Additionally, because these structures are highly interconnected [51], death of a cell in one or the other structures could result in the death of the connecting cells in a process termed transneuronal degeneration. Neurons are highly dependent on continual input from interconnecting cells for survival, meaning if an upstream cell is damaged or dies, so too might the downstream [14]. This is another mechanism that could in part account for some of the infection-independent apoptosis observed in AIR^low^ mice.

## Conclusions

Here we demonstrate that vertically transmitted ZIKV can establish infection in the CNS of postnatal AIR^low^ mice resulting in structural abnormalities. Analysis of multiple possible mechanistic mediators demonstrated cell death, gliosis and altered neural progenitor proliferation in CNS areas involving these abnormalities suggesting these processes may be involved. Interestingly, not all changes were due to direct ZIKV infection in the CNS as developmental gene expression and cerebellar granular layer formation were impacted, albeit to a lesser extent, in IgR mice where virus was not detectable in the brain. Thus, both maternal and neonatal CNS infection appear to impact neurodevelopment during vertically transmitted ZIKV infection.

## Supplementary Information

**Additional file 1: Fig. S1**. ZIKV-infected IgR mice from one litter had elevated neurodegenerating and active-caspase 3 positive cells in the cerebellum but without detectable virus. Representative H&E-stained cerebellar sections from two (a-c) IgR mice (XZ384P4 and XZ384P5) from the same litter showed minimal-to-mild cell death (black arrows). Representative immunofluorescence labeled (d) cerebellar section from XZ384P5 labeled for ZIKV NS5 (green) and active-Caspase 3 (magenta) and Hoechst (grey) showing a small number of active-Caspase positive cells (white arrows and white boxes which correspond to higher magnification images (e-h).

**Additional file 2: Fig. S2**. Peripheral immune cell infiltration into the brain is absent in AIR^low^ mice. A representative section from an AIR^low^ animal labeled by (a) immunofluorescence for ZIKV NS5 (green) and Iba1 (magenta) showing reactive microglia (yellow arrows) and peripheral myeloid cells within blood vessels (white arrows), but no evidence of immune cell infiltrates around blood vessels (black arrows) in the adjacent (b) H&E-stained section. The scale bar in (a) applies to (b). Cell nuclei were labeled with Hoechst in (a, grey).

**Additional file 3: Fig. S3**. mRNA expression of neurodevelopmental genes continues to trend higher in P14 AIR^low^ mice while mRNA specifically expressed by Purkinje cells is significantly decreased. P14 neonatal whole brain from naïve, IgR and AIR^low^ pups was evaluated for RNA expression of (a) Purkinje cell-specific *Pcp2(L7)* and neurodevelopmental genes including (b) *Mcph1*, (c) *Aspm*, (d) *CASC5*, (e) *WDR62*, (f) *CDK5*, and (g) *Cenpj* by qRT-PCR with specific primers. Each symbol indicates an individual brain. The mean of the individually plotted data points for each group is represented by the horizontal black bars. A One-way ANOVA was used to compare RNA expression of (a) *Pcp2(L7)* (F_2, 12_=10.33, p=0.0025), (b) *Mcph1 *(F_2, 12_=2.73, p=0.1056), (c) *Aspm *(F_2, 12_=3.43, p=0.0665), (d) *CASC5 *(F_2, 12_=5.60, p=0.0191), (e) *WDR62 *(F_2, 12_=2.447, p<0.1284), (f) *CDK5 *(F_2, 12_=2.33, p=0.1397), and (g) *Cenpj* (F_2, 12_=1.29, p=0.3116) from samples from each group and a Tukey Multiple Comparisons Test was used to determine significance between groups. **p<0.01, indicates significance between groups. n.s.=not significant.

## Data Availability

All data generated or analyzed during this study are included in this published article [and its supplementary information files].

## Abbreviations

ZIKV: Zika virus
AIR: Anti-IFNAR1 treated, *Rag1*^−^^/^^−^ mice
AIR^low^: Anti-IFNAR1 treated, *Rag1*^−^^/^^−^ mice low infectious dose
IgR: Normal mouse IgG treated, *Rag1*^*−*^^*/*^^*−*^ mice
VTx: Vertical transmission
CNS: Central nervous system
P: Postnatal day
dpi: Days post-infection
PFU: Plaque forming unit
IP: Intraperitoneally
H&E: Hematoxylin and eosin
IHC: Immunohistochemistry
Iba1: Ionized calcium binding protein
GFAP: Glial fibrillary acidic protein
TMEM119: Transmembrane protein 119
EGL: External germinal layer
PCL: Purkinje cell layer
ML: Molecular layer
GL: Granular layer
qRT-PCR: Quantitative real-time PCR
