# Supplementary figures and images for: Zika virus vertical transmission in interferon receptor1-antagonized Rag1−/− mice results in postnatal brain abnormalities and clinical disease
Source: Acta Neuropathol Commun. 2022 Apr 4;10:46. doi: 10.1186/s40478-022-01351-6 (PMC8981715; doi:10.1186/s40478-022-01351-6)

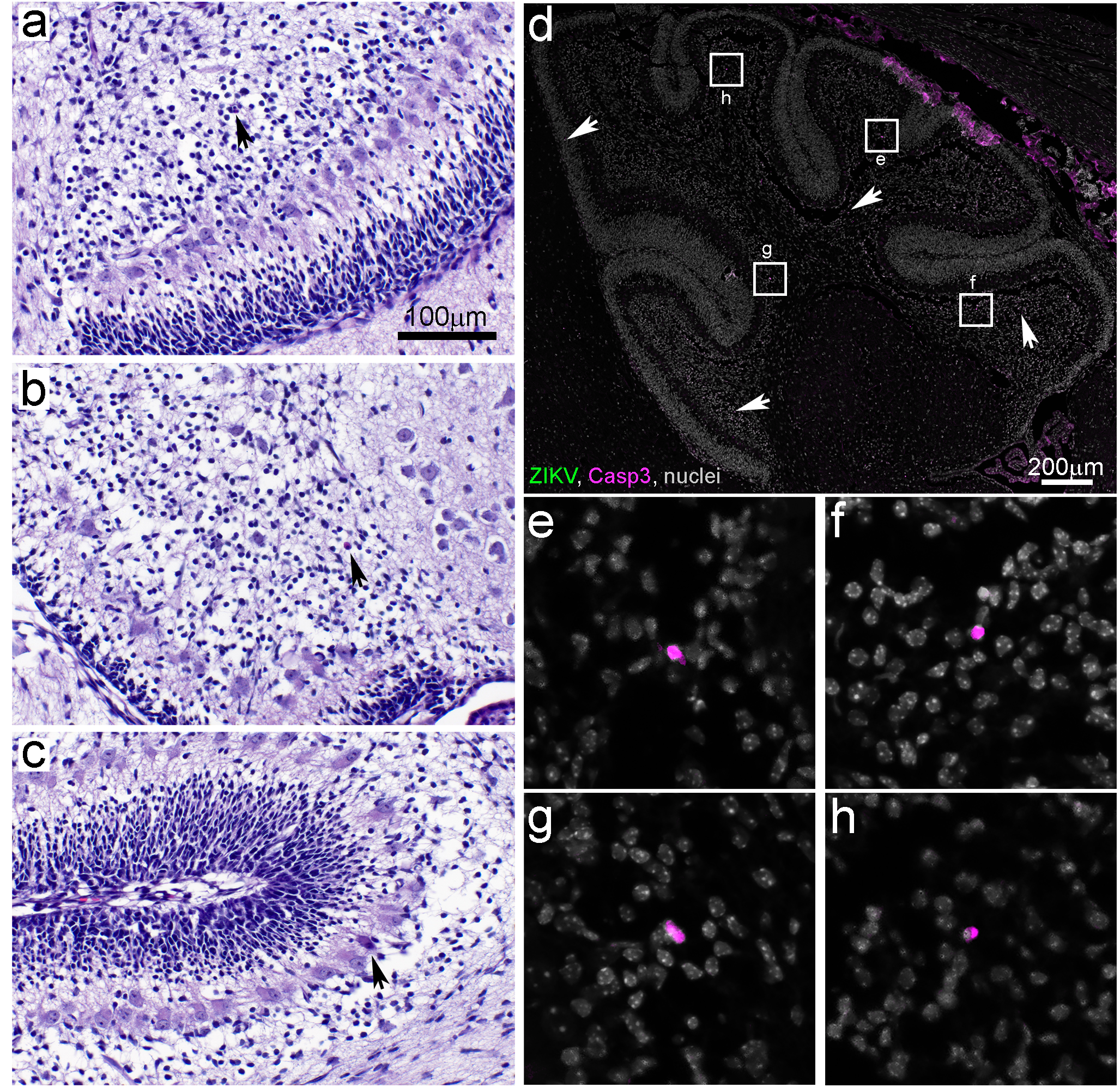

Supplement: Supplementary file 1 — Additional file 1: Fig. S1. ZIKV-infected IgR mice from one litter had elevated neurodegenerating and active-caspase 3 positive cells in the cerebellum but without detectable virus. Representative H&E-stained cerebellar sections from two (a-c) IgR mice (XZ384P4 and XZ384P5) from the same litter showed minimal-to-mild cell death (black arrows). Representative immunofluorescence labeled (d) cerebellar section from XZ384P5 labeled for ZIKV NS5 (green) and active-Caspase 3 (magenta) and Hoechst (grey) showing a small number of active-Caspase positive cells (white arrows and white boxes which correspond to higher magnification images (e-h). [file 40478_2022_1351_MOESM1_ESM.tif]

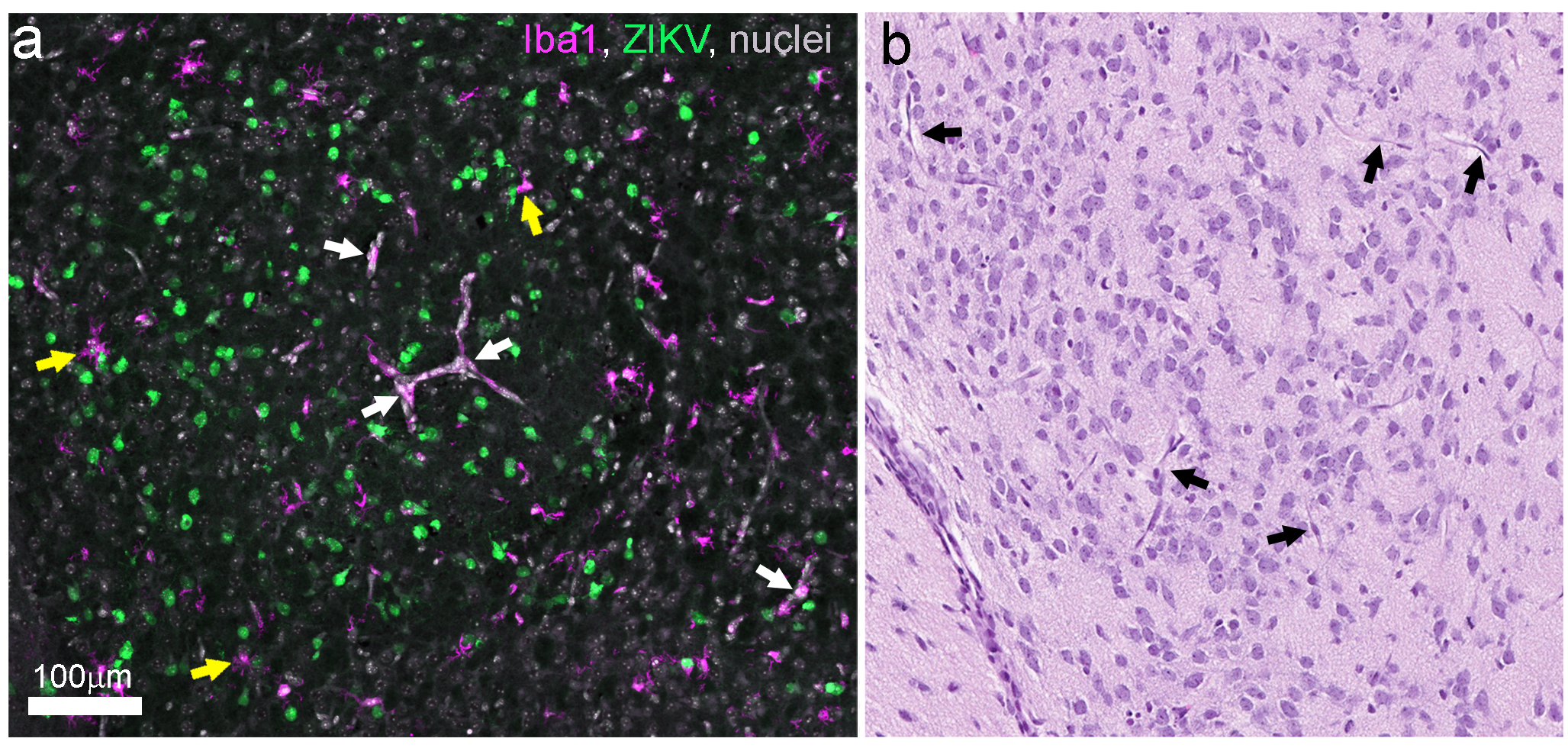

Supplement: Supplementary file 2 — Additional file 2: Fig. S2. Peripheral immune cell infiltration into the brain is absent in AIRlow mice. A representative section from an AIRlow animal labeled by (a) immunofluorescence for ZIKV NS5 (green) and Iba1 (magenta) showing reactive microglia (yellow arrows) and peripheral myeloid cells within blood vessels (white arrows), but no evidence of immune cell infiltrates around blood vessels (black arrows) in the adjacent (b) H&E-stained section. The scale bar in (a) applies to (b). Cell nuclei were labeled with Hoechst in (a, grey). [file 40478_2022_1351_MOESM2_ESM.tif]
